# Morphological and toxicological effects of a deltamethrin-based insecticide on aquatic insect larvae: *Grumichella boraceia* (Trichoptera) as a bioindicator of pyrethroid contamination

**DOI:** 10.1007/s10646-026-03121-9

**Published:** 2026-07-02

**Authors:** Álvaro Domingues Ataide, Laryssa Lemos da Silva, Giovanna dos Santos Pereira, Filipe Schitini Salgado, Dayvson Ayala da Costa, José Eduardo Serrão

**Affiliations:** 1 Department of Entomology, Federal University of Viçosa, Viçosa, Brazil; 2 Department of General Biology, Federal University of Viçosa, Viçosa, Brazil

**Keywords:** Caddisfly, Decis 25EC^®^, ecotoxicology, morphology, pyrethroid

## Abstract

Insect pest control is essential in agriculture to prevent losses and increase productivity. However, excessive insecticide use can negatively impact aquatic ecosystems. Deltamethrin, a widely used pyrethroid insecticide affects non-target insects by compromising the midgut and fat body—organs crucial for digestion, detoxification, and energy storage. Trichoptera larvae are sensitive to pesticides in aquatic environments, making them valuable bioindicators. This study describes the morphology and histopathological effects of a sublethal concentration of a deltamethrin-based insecticide Decis EC25^®^ on the fat body and midgut of *Grumichella boraceia* (Trichoptera) larvae. Larvae were collected and maintained in the laboratory, where the 24-hour LC₅₀ was estimated at 0.0037 µg a.i. L⁻¹, below the residual levels commonly found in aquatic habitats. The larval midgut epithelium consists of columnar digestive cells with an apical brush border and a central ovoid nucleus with decondensed chromatin, alongside goblet-like cells with a cavity, brush border, and basal nucleus. Regenerative cell nests are present at the base of the epithelium, with surrounding circular and longitudinal muscle layers. The fat body is well-developed, containing trophocytes with irregular nuclei rich in decondensed chromatin and basophilic cytoplasm with lipid droplets. While the fat body showed no histopathological alterations after deltamethrin-based insecticide exposure, the midgut presented epithelial disorganization, apical cell protrusions, cytoplasmic vacuolization with abundant spherocrystals, and apocrine secretion. This is the first histological description of the larval midgut and fat body in *Grumichella*, revealing similarities with Lepidoptera larvae. The findings demonstrate that the deltamethrin-based insecticide Decis 25EC^®^ is toxic to *G. boraceia*, impairing its physiology and survival even at sub-residual concentrations.

## Introduction

In agriculture, pest control is essential to ensure economically viable production. Among pests, insects cause the most damage, not only by feeding on crops but also by acting as vectors of plant diseases. Chemical insecticides are the main control method used; however, they pose a risk of contaminating aquatic ecosystems (Dellamatrice and Monteiro 2014). Among insecticides, pyrethroids act by targeting sodium-potassium channels in neurons (Soderlund 2012) and inhibiting the detoxifying enzyme glutathione S-transferase in insects, leading to death (Ribeiro et al. 2022). Deltamethrin, a pyrethroid, has been detected in residual concentrations in soil, plants, and water (Hunt et al. 2016; Reiber et al. 2021; Dellamatrice and Monteiro 2014).

Non-target insects are often affected by insecticide applications, including pollinators, predators, parasitoids, coprophages (Hafsi et al. 2016; Serrão et al. 2022), and aquatic insects (Montaño-Campaz et al. 2022). Among aquatic insects, Trichoptera larvae may build shelters, capture nets, or live freely (Flint et al. 1999; Pes et al. 2005). These larvae are widespread in lakes and rivers, fulfilling diverse ecological roles and feeding strategies, and play a key role in freshwater food webs (Pimentel et al. 2020; Cobo 2005). They are also widely recognized as bioindicators of environmental pollution (Ruiz-García et al., 2022).

Within Trichoptera, the Leptoceridae includes larvae that construct mobile cases from silk and detritus (Tasker and Bilton 2023). This family is globally distributed and includes 46 genera and 1,567 species (Holzenthal and Pes, 2004). The genus *Grumichella* Mueller, 1879 includes several recently described species. Despite growing interest in this group, there are still major gaps in knowledge regarding its biodiversity and biology (Calor et al. 2016, 2024). One of the recently described species, *Grumichella boraceia* Calor and Holzenthal, 2016, has been studied for aspects such as its reproductive system (Costa et al. 2024). However, no studies to date have investigated other key internal structures, such as the midgut and fat body.

The insect midgut is a critical organ for studying insecticide action, as it not only facilitates digestion and nutrient absorption but also serves as a physical and chemical barrier against pathogens and toxic substances (Denecke et al. 2018; Castro et al. 2019). Generally, the insect midgut comprises a simple epithelium with columnar digestive cells involved in digestion and absorption, endocrine cells that produce hormonal peptides, and regenerative cells responsible for epithelial renewal (Caccia et al. 2019). In Lepidoptera, goblet cells may also be present (Terra and Ferreira 2020). In Trichoptera, midgut histology remains poorly documented, with only one species, *Limnephilus stigma* Curtis, 1834 (Limnephilidae), having been studied, revealing columnar digestive cells, cells rich in secretory granules, and regenerative cell nests (Chayka and Farafonova 1980).

In larval insects, nutrients absorbed through the midgut are stored in the fat body as polysaccharides, proteins, and lipids, which serve as energy sources for various physiological functions, including detoxification of xenobiotics such as insecticides (Guedes et al. 2006; Dutra et al. 2019). The fat body is primarily composed of trophocytes, which originate from the endoderm (Paes de Oliveira and Cruz-Landim 2003), and is also poorly studied in Trichoptera (Meier et al. 2000).

Due to their sensitivity to environmental changes caused by insecticides and their measurable physiological responses, caddisfly larvae are widely used in assessments of aquatic ecosystem quality (Ratia et al. 2012). Therefore, studies focused on the morphophysiology, behavior, and genetics of Trichoptera are essential to better understand how these insects respond to environmental stressors (Berra et al. 2006; Yokoyama et al. 2009a, b; Piccardo et al. 2021).

The objective of this study was to describe the morphology of the midgut and fat body of *G. boraceia* larvae and to evaluate the toxicity and histopathological effects of the deltamethrin-based insecticide Decis 25EC^®^ on this aquatic insect.

## Materials and methods

### Insects

Larvae were collected between January and October 2024 at Fazenda Remanso (20°39’ S, 42°27’ W), located in the municipality of Araponga, Minas Gerais, Brazil. Specimens were sampled using granulometric sieves (7.6 cm in diameter, 8 mm mesh size) at multiple points along the river, covering areas from the lowest to the highest elevation, with a total sampling effort of 8 h of active search (Pimentel et al. 2020).

Collected larvae were transported to the Federal University of Viçosa in polystyrene boxes filled with river water. A fine mesh fabric was added to facilitate larval adhesion and reduce stress during transport (Ratia et al. 2012). To prepare for laboratory rearing, stony substrate from the collection site was washed and air-dried according to Villamarín et al. (2022).

In the laboratory, larvae were acclimated in river water for 24 h, then transferred (*n* = 100) to 10 L glass aquariums equipped with 250 L h⁻¹ Hang On filters to ensure adequate water oxygenation. The aquariums were maintained at 20–23 °C. The collected stones were placed at the bottom of the aquariums to simulate the natural riverbed environment.

### Concentration-mortality bioassay

A concentration–mortality bioassay was conducted following the USEPA (1975) and OECD guideline 235 (OECD 2011). Larvae measuring 1–2 cm in length, collected in the field and maintained in the laboratory for 36 h as aforementioned, were used for the test. A commercial formulation of deltamethrin [Decis 25EC^®^; 25 g L⁻¹ of the active ingredient S)-α-cyano-3-phenoxybenzyl (1R,3R)-3-(2,2-dibromovinyl)-2,2-dimethylcyclopropanecarboxylate, 789.4 g L⁻¹ of aromatic hydrocarbons, and 75.6 g L⁻¹ of other ingredients; Bayer CropScience Ltda., São Paulo, Brazil)] was used to prepare a stock solution at a concentration of 25 µg a.i. L⁻¹ in distilled water. The field concentration of deltamethrin, 0.06 µg L⁻¹ reported in a river of the same basin where the insects were collected (Davia et al. 2018) was prepared to conduct a 24 h pilot experiment to verify mortality, showing 100% of mortality. Based on this finding a final series consisting in four concentrations (0.06, 0.045, 0.03, and 0.0015 µg a.i. L⁻¹) were prepared to evaluate acute toxicity and to estimate the lethal concentrations (LC₅₀, LC₇₅, and LC₉₀). For each concentration and the control, 10 larvae were placed in 200 mL of dechlorinated tape water in plastic vials, with four replicates per insecticide concentration and control, totaling 200 individuals. Larval mortality was recorded every 24 h for up to 72 h. During the exposure the insects were not fed and the water kept at 21–23 °C and pH 7.5. Individuals that failed to respond to stimulation with a Pasteur pipette or showed no motility were considered dead and removed from the container. Any abnormal behaviors observed during the assay were also documented. Control groups were maintained under identical conditions but without insecticide exposure. Lethality was assessed using a concentration–response Probit model, following the method described by Finney (1964). Data were assessed for normality with Shapiro-Wilk test and for homoscedasticity with Barlett test, following Probit analysis in the software R-Studio 4.4.0 with 5% of significance.

### Deltamethrin-based insecticide acute exposure

After estimating the lethal concentrations, a separate set of larvae was submitted to acute exposure to the LC₅₀ concentration of deltamethrin-based insecticide, as previously described, for a period of 24 h. Following exposure, surviving larvae were used for histopathological analysis of the midgut and fat body to assess sublethal effects of the insecticide.

### Light microscopy

After 24 h of exposure to the LC₅₀ concentration of the insecticide, larvae from both the treated group (*n* = 5) and the control group (*n* = 5) were used for light microscopy analyses according to Barbosa et al. (2015). Briefly, they were transferred to 2.5% glutaraldehyde in 0.15 M sodium cacodylate buffer (pH 7.2) for 48 h. Larvae were then removed from their cases, rinsed in the same buffer, dehydrated through a graded ethanol series (70%, 80%, 90%, and 95%), and embedded in Leica historesin according to the manufacturer’s instructions. Semi-thin sections (3 μm thick) were stained with hematoxylin (15 min) and eosin (30 s) and examined under a light microscope (Olympus BX60).

### Histochemistry

For histochemical analysis, some unstained histological sections of *G. boraceia* larvae obtained as described above were used to detect proteins and glycoconjugates.

To identify total proteins, sections were stained with mercury–bromophenol blue (Bancroft and Gamble 2008). Samples were immersed in a staining solution composed of 2% acetic acid, 0.05 g bromophenol blue, and 1.5 g mercuric chloride for 2 h and 15 min. They were then washed with 0.5% acetic acid for 10 min, followed by running tap water for 15 min, and analyzed under a light microscope (Olympus BX60).

To detect neutral polysaccharides, the periodic acid–Schiff (PAS) technique was used (Bancroft and Gamble 2008). Sections were treated with 0.4% periodic acid for 30 min, rinsed with distilled water, and incubated in Schiff’s reagent for one hour in the dark. After staining, the samples were washed under running tap water for 30 min and examined using the same microscope.

## Results

### Concentration-mortality

The concentration–mortality model was statistically adequate (*P* > 0.05), confirming the toxicity of the deltamethrin-based insecticide to *G. boraceia* larvae measuring 1–2 cm in length and enabling the estimation of lethal concentrations (Table 1). The LC₅₀ value after 24 h of exposure was 0.0037 µg a.i. L⁻¹.

**Table 1:** Lethal concentrations (LC; µg L^-1^) of deltamethrin estimated for *Grumichela boraceia* larvae (Trichoptera: Leptoceridae) at different exposure times obtained by Probit analysis (*df* = 13)

Time (h)	LC_50_	LC_75_	LC_90_	Slope ± sd	χ2
	(95% CI)*	(95% CI)*	(95 CI%)*		
24	0.0037	0.0097	0.0149	116.49 ± 30.50	2.808
	(0.0013–0.0054)	(0.0076–0.011)	(0.011–0.019)		
48	0.0027	0.0136	0.0233	62.22 ± 28.67	3.537
	(0.0020–0.0083)	(0.0075–0.019)	(0.009-0.038)		
72	0.0008	0.0137	0.0253	52.40 ± 28.7	3.392
	(0.0007–0.0092)	(0.0063–0.021)	(0.006–0.044)		

* 95% confidence interval

During the bioassay, larvae exposed to the four insecticide dilutions (0.06, 0.045, 0.03, and 0.0015 µg a.i. L⁻¹), after 24 h, exhibited abnormal behavior, notably the abandonment of their cases. In contrast, control group larvae remained within their shelters and tended to aggregate. Treated larvae showed reduced motility and a diminished response to mechanical stimuli compared to the control group.

### Midgut

**Fig. 1:**
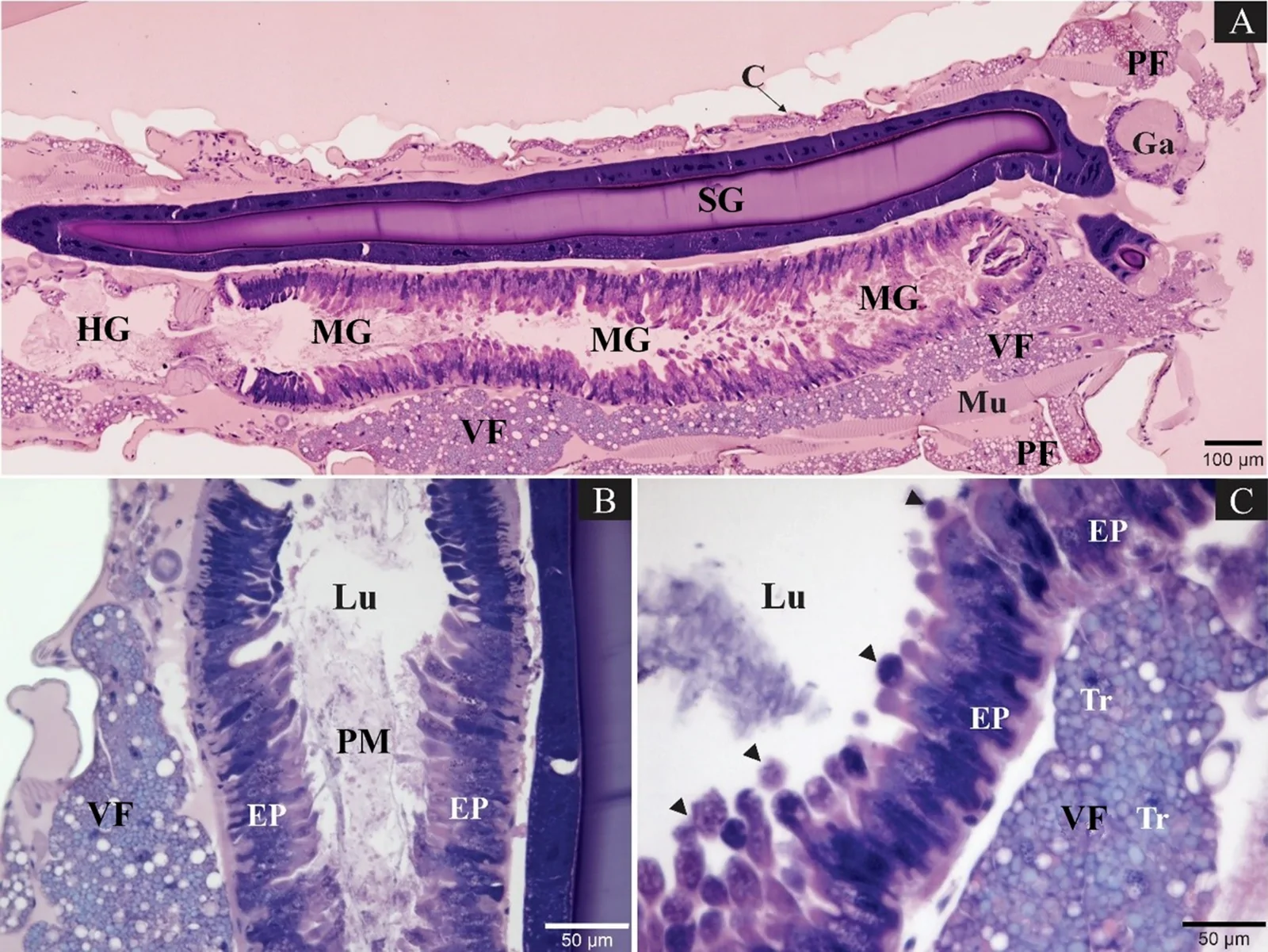
Light micrographs of *Grumichella boraceia* larvae in the control group. (**A**) General view showing the body cuticle (C), midgut (MG), short hindgut (HG), well-developed salivary gland (SG), visceral fat body (VF), parietal fat body (PF), muscle (Mu), and ganglia (Ga). (**B**) Midgut with simple columnar epithelium (EP) and peritrophic matrix (PM) in the lumen (Lu). (**C**) Detail of the midgut epithelium (EP) showing the occurrence of apocrine secretion (arrowheads). Note closely associated visceral fat body (VF) with trophocytes (Tr)

**Fig. 2:**
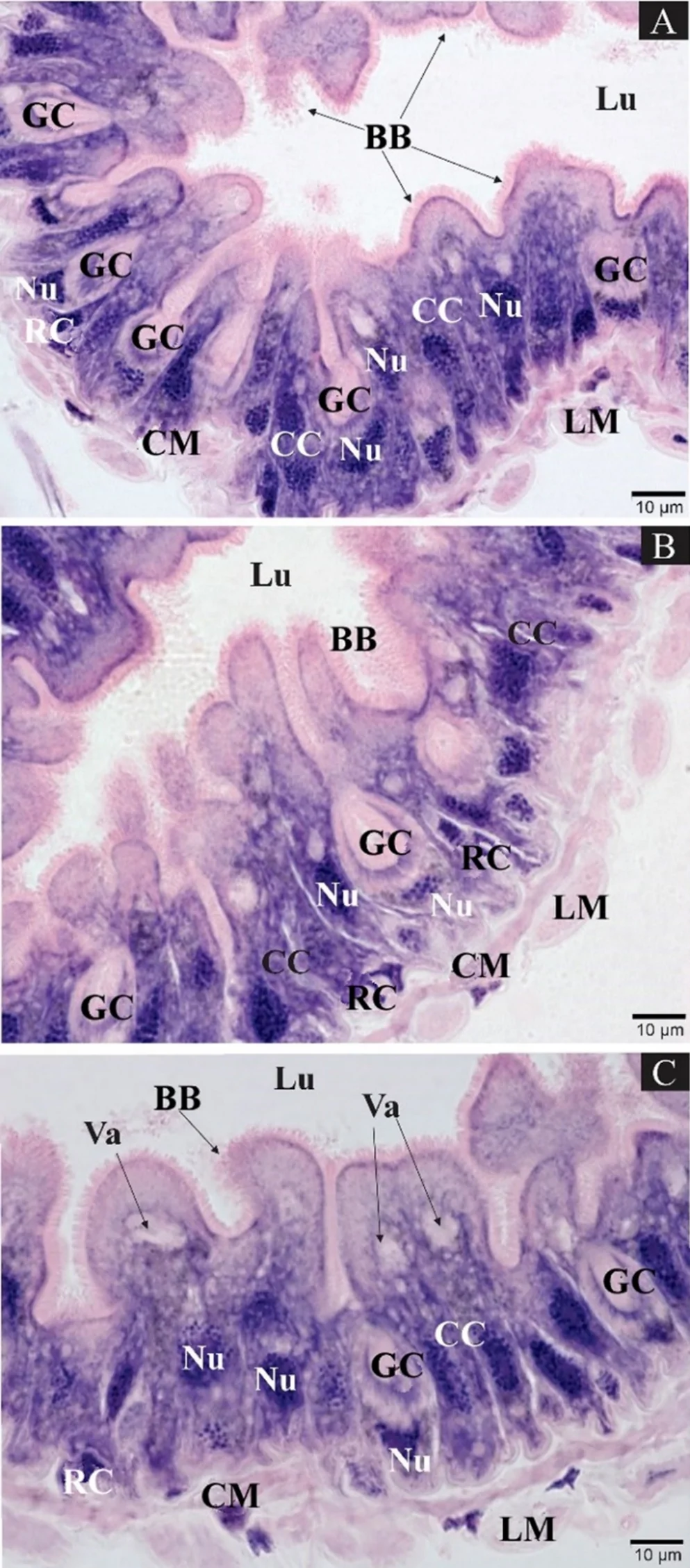
Light micrographs of the midgut of *Grumichella boraceia* larvae in the control. (**A**) Epithelium showing the presence of goblet-like cells (GC), columnar digestive cells (CC) with brush border (BB) and nests of regenerative cells (RC). (**B**) Detail of columnar digestive cells (CC) with acidophilic apical and basophilic median-basal cytoplasm, goblet-like cells with well-developed cavity (GC) and basal nucleus (Nu). Note the longitudinal (LM) and circular (CM) muscle layers. (**C**) Epithelium showing columnar digestive cells (CC) with median oval nuclei (Nu) and cytoplasm with well-developed vacuoles (Va)

In *G. boraceia*, the digestive tract features a prominently developed midgut located in the median region of the body, running parallel to the elongated salivary gland (Fig. 1A). The midgut wall is composed of a single-layered columnar epithelium, an inner circular and an outer longitudinal muscle layer (Figs. 1A–C). A peritrophic matrix was observed into the midgut lumen (Fig. 1B). In some larvae, apocrine secretion was detected in the median region of the midgut epithelium (Fig. 1C). The epithelium contains columnar digestive cells, goblet-like cells, and nests of regenerative cells (Fig. 2A).

Columnar digestive cells are the most abundant, with oval nuclei located in the median portion of the cell and rich in decondensed chromatin (Fig. 2). Their apical surface displays a well-developed brush border (Fig. 2A and B). The apical cytoplasm is acidophilic and contains large vacuoles and granules, whereas the median to basal regions exhibits basophilic cytoplasm with smaller vacuoles (Fig. 2B and C).

Goblet-like cells are less numerous and are identified by an apical invagination forming an enlarged cavity with a brush border (Fig. 2B and C). The nucleus is basal and contains predominantly decondensed chromatin (Fig. 2B and C).

Regenerative cells occur in small clusters at the base of the epithelium and do not extend to the lumen. They are characterized by prominent nuclei with condensed chromatin (Fig. 2).

**Fig. 3:**
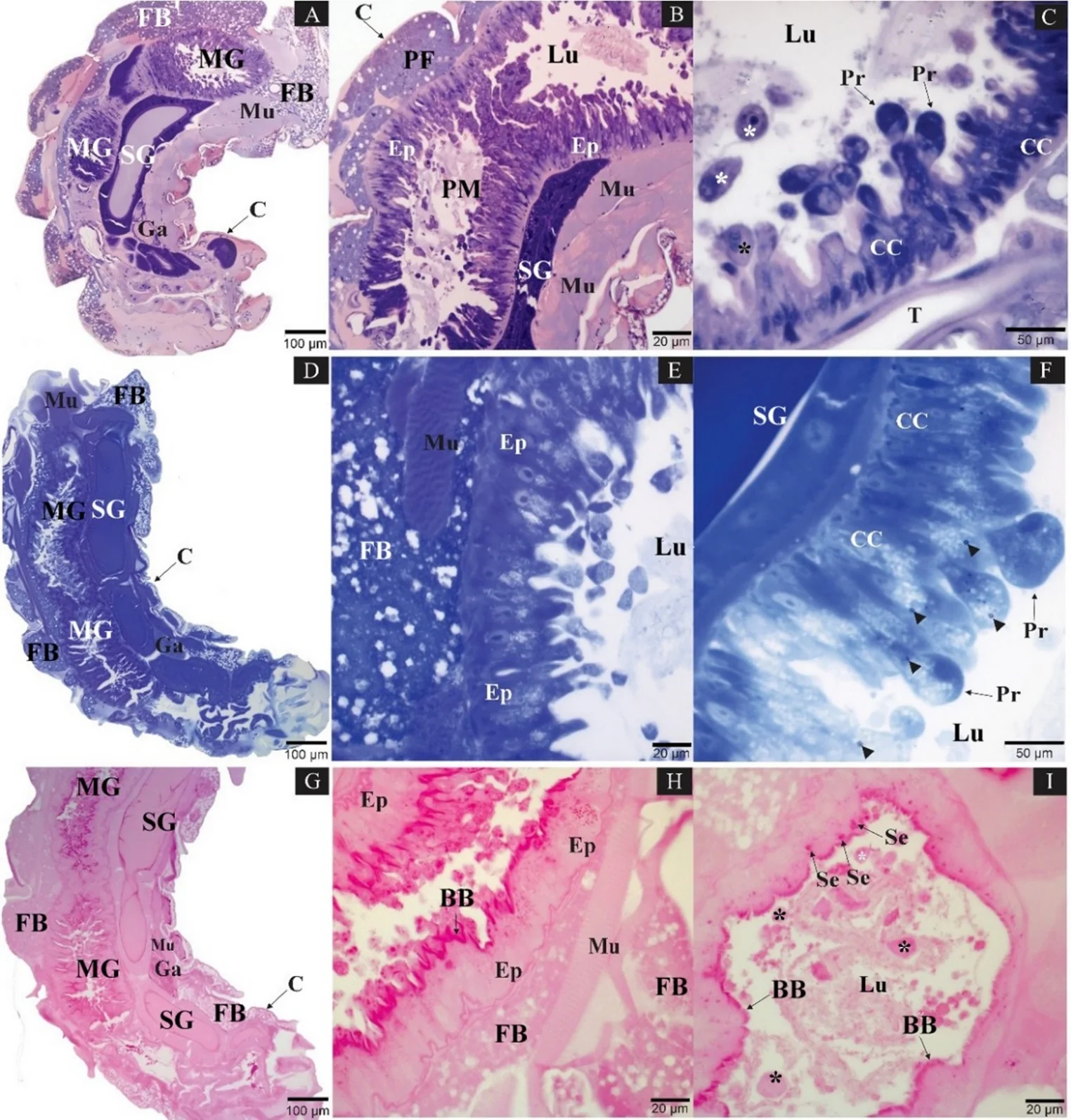
Light micrographs of *Grumichella boraceia* larvae exposed to the deltamethrin-based insecticide Deis 25EC^®^ (0.0037 µg a.i. L⁻¹) for 24 h. **A** - **C**) Longitudinal sections showing the midgut (MG) with columnar epithelium (Ep), lumen (Lu) with peritrophic matrix (PM), salivary glands (SG), muscle (Mu) and body cuticle (C). Note apical protrusions (Pr) and cell fragments (asterisks) released to the midgut lumen. Hematoxylin and eosin stained. HE staining. **D** - **F**) Longitudinal sections showing midgut epithelium with apical protrusions (Pr) and vacuoles (arrowheads). Mercury-bromophenol test. **G** – **I**) Longitudinal sections showing midgut epithelium with brush border (BB), secretory vesicles (Se) and cell fragments (asterisks). PAS histochemical test. CC – columnar digestive cell. FB - fat body, Ga – ganglia.

After 24 h of exposure to the LC₅₀ concentration of the deltamethrin-based insecticide, all the five *G. boraceia* larvae analyzed showed midgut damage compared to controls. This included epithelial disorganization, apical cytoplasmic protrusions of columnar digestive cells, some released as fragments into the lumen, and intense cytoplasmic vacuolization (Figs. 3A–C).

Histochemical analysis using mercury–bromophenol blue and PAS tests showed positive reactions for proteins and glycoconjugates, respectively, in the midgut epithelium of both control and deltamethrin-exposed larvae. Mercury–bromophenol test was uniformly positive throughout the epithelium, including the apical protrusions, though vacuoles remained unstained (Figs. 3D–F). The PAS reaction revealed strongly positive vesicles in the apical cytoplasm and in the brush border of columnar cells in both control and treated groups (Figs. 3G–I).

### Fat body

**Fig. 4:**
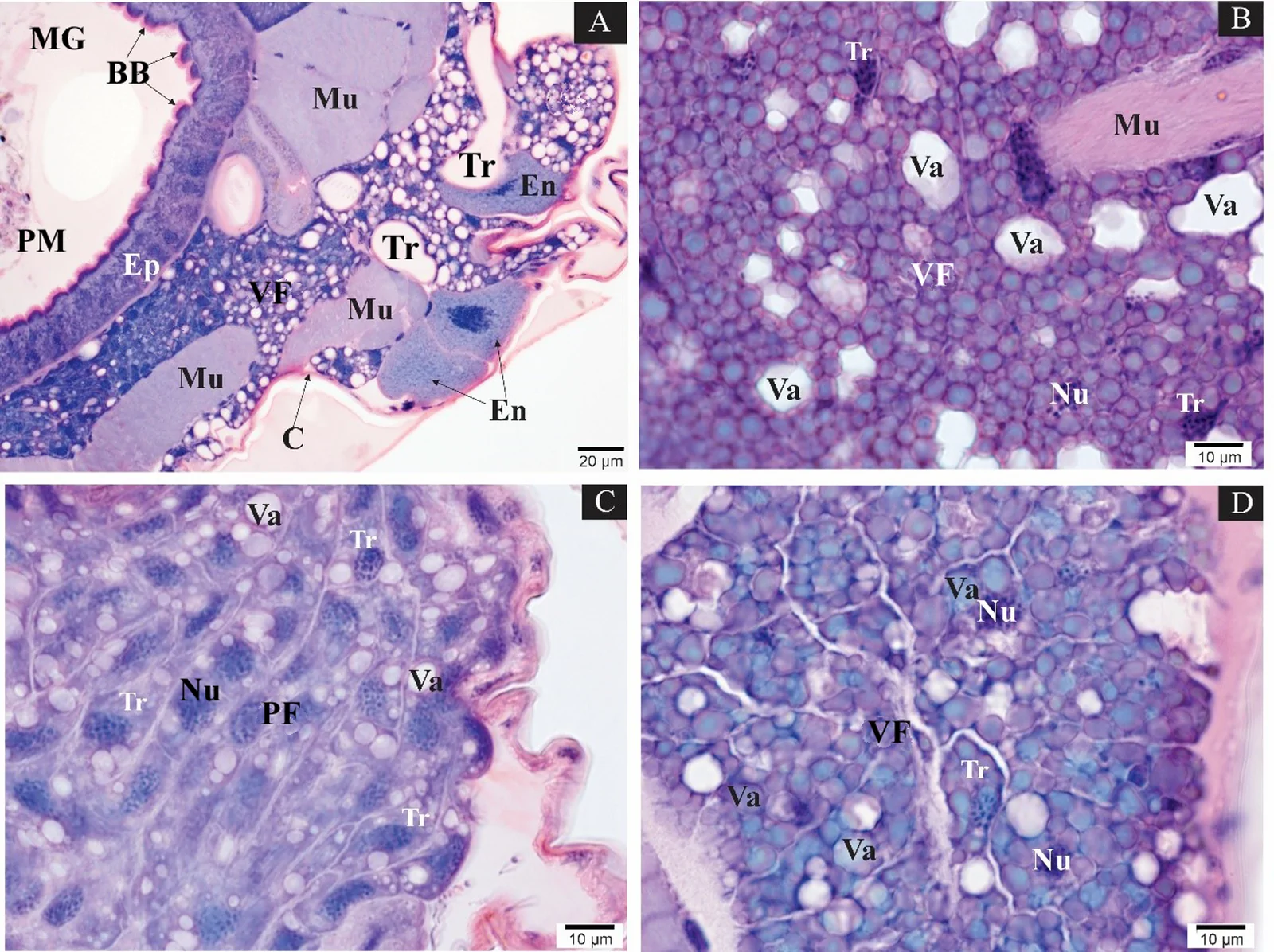
Light micrographs of *Grumichella boraceia* larva. (**A**) Cross-section showing the midgut (MG), visceral fat body (VF) and oenocytes (En). (**B**) Visceral fat body (VF) with trophocytes (Tr) showing large vacuoles (Va) and well-developed nucleus (Nu). (**C**) Parietal fat body (PF) with well-developed trophocytes (Tr) with small vacuoles (Va) and nucleus (Nu). (**D**) Visceral fat body (VF) showing some trophocytes (Tr) with nucleus rich in condensed chromatin (Nu). BB - brush border; C – body cuticle, Ep = epithelium; Mu = muscle; PM = peritrophic matrix; Tr = trachea. A–B) control larvae. C-D) larvae exposed to the deltamethrin-based insecticide Deis 25EC^®^ (0.0037 µg a.i. L⁻¹) for 24 h

In *G. boraceia* larvae, the fat body is differentiated into parietal and visceral regions based on location and structural organization (Fig. 4A). The parietal fat body is situated in the peripheral abdominal region, adjacent to the integument (Figs. 4C). It has a sheet-like arrangement, composed of thin layers of cells containing numerous small vacuoles (Fig. 4C). The visceral fat body is located near the midgut, and is composed of cells with large, well-developed vacuoles (Fig. 4B and D). Fat body cells (trophocytes) exhibit irregularly shaped nuclei with predominantly decondensed chromatin (Fig. 4).

Larvae exposed to the LC₅₀ concentration of deltamethrin-based insecticide for 24 h showed no morphological differences in either the parietal or visceral fat body when compared to control individuals.

To investigate potential physiological alterations caused by insecticide exposure, the fat body was subjected to histochemical tests for the detection of neutral polysaccharides and total proteins. Both the periodic acid-Schiff (PAS) and mercury–bromophenol blue stains revealed similar positive reactions in treated and control larvae, indicating comparable distributions of glycoconjugates and proteins.

## Discussion

The estimated LC₅₀ value of deltamethrin-based insecticide Decis 25EC^®^ for *G. boraceia* larvae after 24 h of exposure was 0.0037 µg a.i. L⁻¹, a concentration 16.2-folds lower than the field-relevant residual level of 0.06 µg L⁻¹ previously reported in the same river basin (Davia et al. 2018). This highlights the high sensitivity of *G. boraceia* to deltamethrin and raises concerns about the environmental safety of this insecticide in aquatic ecosystems, especially those near agricultural areas.

When compared to other aquatic arthropods considered sensitive to pyrethroids, *G. boraceia* displays even lower LC₅₀ values. For example, *Callibaetis radiatus* (Ephemeroptera: Baetidae) has an LC₅₀ of 0.60 µg a.i. L⁻¹ for 24-hour exposure to deltamethrin (Gutierrez et al., 2016), and *Chaoborus obscuripes* (Diptera: Chaoboridae), a species known for its sensitivity, exhibits LC₅₀ values of 0.027 µg a.i. L⁻¹ and 0.075 µg a.i. L⁻¹ after 48 and 96 h of exposure to lambda-cyhalothrin, respectively (Schroer et al. 2004). These values, although low, remain higher than the LC₅₀ observed here for *G. boraceia*, indicating its vulnerability to pyrethroids.

Our findings of the high sensitivity suggests that *G. boraceia* may serve as an effective bioindicator of freshwater contamination by insecticides, supporting the inclusion of this species in ecological risk assessments and biomonitoring programs focused on freshwater ecosystems.

During sublethal exposure to deltamethrin-based insecticide Decis 25EC^®^, *G. boraceia* larvae exhibited shelter abandonment behavior. This response, which may represent an attempt to escape a contaminated environment, was similarly reported in *Brachycentrus americanus* larvae (Trichoptera: Brachycentridae) exposed to the pyrethroid esfenvalerate (Johnson et al. 2008). However, this behavior increases the larvae vulnerability to predation (Palmquist et al. 2008), suggesting that even larvae that survive deltamethrin exposure may face further ecological risks, such as increased mortality from predators.

In addition to case abandonment, *G. boraceia* larvae exposed to the deltamethrin-based insecticide failed to exhibit the natural assembling behavior observed in control larvae. This alteration in behavior is similar to reported for other insects, such as *Plutella xylostella* (Linnaeus, 1767) larvae (Lepidoptera: Plutellidae) exposed to permethrin (Hoy et al. 1991). Furthermore, the reduced motility and diminished response to stimuli observed in deltamethrin-exposed *G. boraceia* larvae were comparable to those described for *Spodoptera frugiperda* J. E. Smith, 1797 larvae (Lepidoptera: Noctuidae) when exposed to insecticides like pyrethroids (Gist and Pless 1985).

Like other Trichoptera, larvae of *G. boraceia* possess an alimentary canal where the midgut is the larger organ (Sangpradub and Giller 1994). In larvae of *Wormaldia mediana* McLachlan, 1878 (Trichoptera: Philopotamidae), the midgut epithelium is composed of columnar (digestive) cells responsible for secreting the peritrophic matrix (Corallini 2011). This characteristic is also observed in *G. boraceia* larvae, which exhibit a peritrophic matrix in the lumen, and the epithelium shows apocrine secretion. The peritrophic matrix is produced along the midgut in larvae of the predator *Ceraclea fulva* (Rambur, 1842) (Trichoptera: Leptoceridae) and serves the role of mechanically protecting the gut epithelium (Corallini 2011). Although *C. fulva* is a predator and *G. boraceia* is a scraper, the similar presence of the peritrophic matrix suggests a shared protective function in the midgut, although the species exhibit different feeding behaviors.

In the midgut epithelium of *G. boraceia*, digestive cells with a well-developed apical brush border are observed, along with regenerative cells and, in lower abundance, goblet-like cells. This cellular composition of the midgut epithelium, comprising columnar, goblet, and regenerative cells, has also been described in larvae of the caddisfly *L. stigma* (Chayka and Farafonova 1980), although the goblet-like cells appear to differ morphologically from those observed in *G. boraceia*. Similarly, caterpillars Lepidoptera exhibit comparable midgut cellular organization (Terra and Ferreira 2020; Castro et al. 2021; Vinha et al. 2021; Lima et al., 2023).

In Lepidoptera, goblet cells are involved in the active transport of potassium from the hemolymph into the midgut lumen, an adaptation associated with their potassium-rich diet (Flower and Filshie 1976; Azuma et al. 1991). Similar cells are present in *G. boraceia* larvae, identifiable by an apical invagination that forms a large cavity with a brush border, a morphology resembling that described in lepidopteran caterpillars (Gomes et al. 2013). However, the precise function of these cells in Trichoptera remains unclear, and further studies are necessary to determine their physiological role in caddisfly larvae.

The presence of goblet cells in the midgut is a morphological trait observed exclusively in Lepidoptera and Trichoptera, both of which belong to the Amphiesmenoptera clade. This feature may represent a synapomorphy of the group, supporting the current phylogeny that recognizes these orders as sister groups (Mey et al. 2016). Moreover, goblet cells are absent in Antliophora, the sister clade to Amphiesmenoptera, which includes Diptera, Mecoptera, and Siphonaptera (Beutel et al. 2011). This distribution reinforces the hypothesis that the presence of goblet cells is an evolutionary novelty shared exclusively by the common ancestor of Amphiesmenoptera. The presence of both parietal and visceral fat bodies in *G. boraceia* is a common feature among many insects, including Lepidoptera, the sister group of Trichoptera. These structures are located in distinct regions of the body and exhibit different cellular arrangements (Haunerland and Shirk 1995), characteristics also observed in *G. boraceia*.

Trophocytes are distributed throughout the fat body of *G. boraceia* larvae, with a higher concentration in the parietal region, similar to what has been reported for *Anticarsia gemmatalis* larvae (Carvalho et al. 2013). These cells are likely involved in glycogen synthesis, storage, and metabolism, as described for *Bombyx mori* (Linnaeus, 1758) (Lepidoptera: Bombycidae) (Pak et al. 2025) and other holometabolous larvae (Roma et al. 2006).

Larvae of *G. boraceia* exposed to the deltamethrin-based insecticide Decis 25EC^®^ exhibited damage to the midgut epithelium, including increased vacuolization, apical cell protrusions, and the release of cell fragments into the lumen. Similar histopathological alterations have been reported in the midgut of Lepidopteran caterpillars, such as *S. frugiperda* exposed to the same insecticide (Vinha et al. 2021), A. *gemmatalis* treated with squamocin (Fiaz et al. 2018a), tebufenozide (Fiaz et al. 2018a, b b), chlorantraniliprole (Castro et al. 2021), and abamectin (Lima et al. 2024). Comparable damage has also been observed in hemimetabolous insects, including *Callibaetis radiatus* (Navás, 1920) (Ephemeroptera: Baetidae) exposed to deltamethrin (Gutiérrez et al. 2016) and *Podisus nigrispinus* (Dallas, 1851) (Heteroptera: Pentatomidae) exposed to spinosad (Santos-Junior et al. 2020). These findings suggest that various pesticides may induce similar histopathological effects in the midgut across diverse insect taxa. The commercial formulation of insecticides includes some adjuvants for improvement of the active ingredient efficiency. Those compounds are generally classified as inert, however, some studies describe lethal and sublethal effects of them on non-target insects (Fine et al. 2017; Shannon et al. 2023 b; Straw and Brown 2021). Therefore, assessing the effects of pesticide formulation on individuals can provide insights into how pesticides affect the biological aspects of the non-target insects such as aquatic ones.

Compared to control larvae, an increased abundance of apocrine secretion was observed in the midgut epithelium of *G. boraceia* exposed to Decis 25EC^®^, which may reflect a detoxification response of the organ to the insecticide. Enhanced apocrine secretion has been reported in the midgut of various insect species exposed to pesticides and is often associated with a potential detoxification mechanism (Martínez et al. 2018; Santos-Junior et al. 2020; Lima et al. 2024; Reis et al. 2024), as these secretions may contain enzymes involved in xenobiotic metabolism (Grella et al. 2019). Therefore, the increased apocrine activity observed in *G. boraceia* may represent a physiological response to mitigate the toxic effects of the deltamethrin-based insecticide.

Moreover, damages in the goblet-like cells found in *G. boraceia*, that in Lepidoptera play some role in generation of electrochemical gradient in the lumen for nutrient absorption (Terra et al. 2019) and detoxification (Gomes et al. 2013) may affect ionic homeostasis and nutrient absorption in the midgut with potential to compromise the caddisfly larvae fitness.

The midgut of *G. boraceia* larvae showed a strong positive reaction for carbohydrates in the brush border and in some vesicles within the apical cytoplasm. In both control and insecticide-exposed groups, mercury bromophenol staining indicated the presence of proteins. However, in larvae exposed to deltamethrin, some vacuoles lacked protein staining and may correspond to spherocrystals. The presence of spherocrystals has been associated with detoxification or osmoregulatory processes (Batista et al. 2023), as observed in *Polistes dominula* (Christ, 1791) (Hymenoptera: Vespidae) workers exposed to heavy metals (Polidori et al. 2018) and in *Apis mellifera* Linnaeus, 1758 following exposure to teflubenzuron (Oliveira et al., 2024). Therefore, the occurrence of spherocrystals in the columnar digestive cells of *G. boraceia* larvae exposed to deltamethrin likely reflects a cellular response involved in mitigating the insecticide’s toxic effects.

*Grumichella boraceia* larvae exposed to Decis 25EC^®^ did not exhibit histopathological damage in the fat body, in contrast to findings for *Caraeochrysa claveri* (Navás, 1911) (Neuroptera: Chrysopidae) (Scudeler et al. 2022) and *Melipona scutellaris* Latreille, 1811 (Hymenoptera: Meliponini) (Farder-Gomes et al. 2024), both of which showed fat body damage following exposure to the insecticides pyriproxyfen and glyphosate, respectively. It is therefore plausible to suggest that the sublethal concentration of the deltamethrin-based insecticide used in the present study may have been detoxified by the fat body, as reported for *Culex quinquefasciatus* Say, 1823 (Diptera: Culicidae) larvae exposed to ivermectin (Alves et al., 2004) and *A.mellifera* treated with imidacloprid (Carneiro et al. 2023).

Although aquatic insect larvae (Antwi and Reddy 2015) and the midgut are not the primary targets of deltamethrin that mainly acts on muscle and nerve cells (Soderlund 2012), the observed histopathological damage in the midgut suggests that *G. boraceia* is sensitive to this insecticide, which is widely used in agriculture.

This study provides the first detailed description of the midgut and fat body morphology in Trichoptera larvae of the genus *Grumichella*. The findings show that the larval morphology of this genus is consistent with that reported for other, albeit few, studied Trichoptera species, featuring a midgut epithelium composed of columnar, goblet-like, and regenerative cells. The fat body is organized into parietal and visceral regions and is composed primarily of trophocytes. Additionally, the results demonstrate that the deltamethrin-based insecticide Decis 25EC^®^ is toxic to *G. boraceia* larvae, as evidenced by histopathological alterations in the midgut and behavioral changes that may compromise larval survival.

## Data Availability

Data will make available upon request for corresponding author.

## Declarations

### Use of generative AI

During the preparation of this work, ChatGPT, AI, was used to improve the language of the manuscript. The authors thoroughly reviewed all content and take full responsibility for the content of the published article.
